# The Effect of Preoperative Melatonin on Nuclear Erythroid 2-Related Factor 2 Activation in Patients Undergoing Coronary Artery Bypass Grafting Surgery

**DOI:** 10.1155/2013/676829

**Published:** 2013-04-08

**Authors:** Shaghayegh Haghjooy Javanmard, Amin Ziaei, Saeid Ziaei, Ehsan Ziaei, Mohsen Mirmohammad-Sadeghi

**Affiliations:** ^1^Department of Physiology, Physiology Research Centre, Isfahan University of Medical Sciences, Hezar Jerib Avenue, Isfahan 73461-8174, Iran; ^2^Medical Student Research Center, Medical School, Isfahan University of Medical Sciences, Isfahan 73461-8174, Iran; ^3^Students' Research Committee, Shahid Beheshti University of Medical Sciences, Tehran, Iran; ^4^Medical Student Research Center, Medical School, Shahrekord University of Medical Sciences, Shahrekord, Iran; ^5^Department of Cardiac Surgery, Isfahan Medical School, Isfahan University of Medical Sciences, Isfahan 73461-8174, Iran

## Abstract

During and after coronary artery bypass grafting (CABG), oxidative stress occurs. Finding an effective way to improve antioxidant response is important in CABG surgery. It has been shown that patients with coronary heart disease have a low Melatonin production rate. The present study aimed to investigate the effects of Melatoninon nuclear erythroid 2-related factor 2(Nrf2) activity in patients undergoing CABG surgery. Thirty volunteers undergoing CABG were randomized to receive 10 mg oral Melatonin (Melatonin group, *n* = 15) or placebo (placebo group, *n* = 15) before sleeping for 1 month before surgery. The activated Nrf2 was measured twice by DNA-based ELISA method in the nuclear extract of peripheral blood mononuclear cells of patients before aortic clumps and 45 minutes after CABG operation. Melatonin administration was associated with a significant increase in both plasma levels of Melatonin and Nrf2 concentration in Melatonin group compared to placebo group, respectively (15.2 ± 4.6 pmol/L, 0.28 ± 0.01 versus 1.1 ± 0.59 pmol/L, 0.20 ± 0.07, *P* < 0.05). The findings of the present study provide preliminary data suggesting that Melatonin may play a significant role in the potentiation of the antioxidant defense and attenuate cellular damages resulting from CABG surgery via theNrf2 pathway.

## 1. Introduction

Myocardial ischemia-reperfusion injury (IRI) represents a clinically critical problem associated with coronary artery bypass surgery (CABG) [1, 2]. Reactive oxygen species, including superoxide radical, hydroxyl radical, and hydrogen peroxide are considered to increase during reperfusion of the heart following ischemia [2]. Systemic increase of hydrogen peroxide and lipid peroxidation products has been shown to occur during CABG operation [3]. The contact of the circulating blood to nonphysiological surfaces during CABG may be another potential source of oxidative stress [4]. Cell death secondary to IRI occurs during the first minutes after restoration of blood flow [5].

 Despite improvements in anesthesia management, surgical technique, and postoperative care, CABG with cardiopulmonary bypass is associated with oxidative stress so it is important to reduce the effect of reactive oxygen species during CABG and find an effective way to improve antioxidant response against IRI [6].

Melatonin (*N*-acetyl-5-methoxytryptamine) is synthesized from tryptophan and secreted principally by the pineal gland [7, 8]. It has an endogenous circadian rhythm of secretion induced by the suprachiasmatic nuclei of the hypothalamus [9]. In mammals, Melatonin is present in almost all tissues, with or without the Melatonin receptors, because it acts both as a hormone and an antioxidant [10].

Melatonin is a highly lipophilic molecule that crosses cell membranes to easily reach subcellular compartments including mitochondria [11]. It stabilizes mitochondria inner membranes [12] and reduces electron leakage and generation of free radicals [13]. Furthermore, Melatonin has potent direct peroxyl radical-scavenging activities [14].

The efficacy of Melatonin as an antioxidant agent relates to its direct actions in scavenging free radicals, and also its potential ability to enhance the activities of a variety of antioxidative enzymes, for instance, its stimulatory effects on the synthesis of glutathione (which is also an important antioxidant) [14]. Melatonin is several times more effective in scavenging the extremely toxic hydroxyl radical than glutathione and mannitol and twice as efficient as vitamin E in detoxifying the peroxyl radical [12, 13].

Several recent experimental studies have linked the beneficial effects of Melatonin in ischemia-reperfusion injuries to nuclear factor (erythroid-derived 2)-like 2, also known as NFE2L2 or Nrf2 pathway activation [15, 16]. Nrf2 is a transcription factor which is responsible for the regulation and management of many antioxidant response genes in cells through binding with DNA antioxidant response element (ARE) [17]. Under normal conditions, Nrf2 is found mainly sequestered in the cytoplasm, tethered by a protein called Keap1; several stimuli can translocate Nrf2 from the cytoplasm to the nucleus. After binding to ARE, Nrf2 transactivates the expression of a group of cytoprotective enzymes, such as heme oxygenase-1 (HO-1), NAD(P)H:quinone oxidoreductase-1 (NQO1), and glutathione S-transferase *α*-1 (GST-*α*1) [18]. Nrf2-ARE pathway is considered to be a main protector and reported to play an important role in ischemia-reperfusion injury [19]. So, it can be considered as a master regulator of antioxidant defense pathway.

The collective results showed that patients undergoing CABG are at risk of free radical damage. Furthermore, it has been shown that Melatonin secretion is reduced in coronary heart disease (CHD) [9, 20]. Melatonin administration against these conditions are due to its direct free radical scavenger activity.

In regard to the beneficial effects of Melatonin to induce activation of Nrf2 as an oxidative stress-sensing guarding regulator leading to expression of important ARE-driven genes such as NQO1, HO-1, SOD2, and GST and, in finding an effective clinical strategy for abrogating reperfusion injury, the purpose of this study was to investigate the potential protective effect of Melatonin against IRI in CABG surgery through the activation of Nrf2 as a critical transcription factor for antioxidant defense management at cellular level.

## 2. Materials and Methods

**Figure 1:**
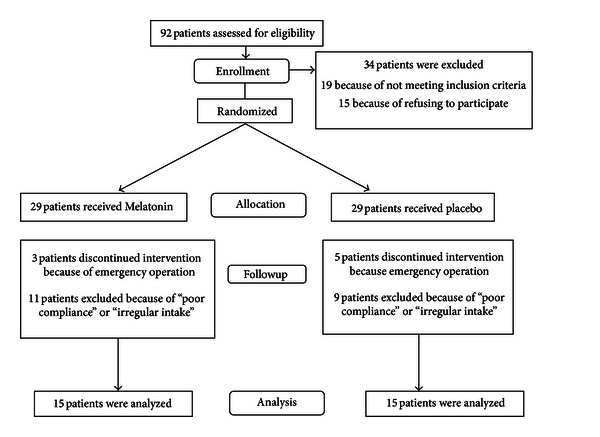
flow diagram of patients participation in the study.

Forty volunteers who were undergoing elective CABG were enrolled in a randomized triple-blind placebo-controlled study. After obtaining approval from the ethics committee of Isfahan University of Medical Sciences and written informed consent, we excluded from the study patients that need emergency operation and have not enough time to use tablets for 1 month, elderly patients (>75 years), reoperations, patients with coexisting renal insufficiency, patients with serious pulmonary disease, those with prior stroke or significant cerebrovascular disease, and patients with an ejection fraction <40%. Patients undergoing CABG with concomitant heart valve repair or replacement, resection of a ventricular aneurysm, or other surgical procedures were also excluded. We included in the study 52 patients who were to undergo elective CABG. The patients were assigned into 2 groups randomly. The patients in group I (*n* = 15) underwent CABG with administration of 10 mg tablet (Melatonin, Nature Made, CA, USA) (Melatonin group) before sleeping for 1 month, and the patients in Group II (*n* = 15) underwent their operations with the usage of placebo in the same course. Flow diagram of patients participation in the study is shown in Figure 1.

According to the previous studies, the effective dosage of Melatonin to reduce oxidative stress related to surgical procedures was 10 mg/kg [21–23].

Patients were allocated in Melatonin or placebo group, using a random number table, to receive either 10 mg oral Melatonin (Melatonin, Nature Made, CA, USA) or placebo 1 month before surgery approximately 1 hour before sleeping. Blinding and randomization were performed by two investigators not involved in the patients' evaluations.

All of the operations in each group started at the same time, and all of the patients in the study underwent their operations with the same technique and by the same surgical team. The operations started regularly at 1–6 PM. All of the patients received the same cardiac drug regimen before the operation, generally angiotensin-converting enzyme inhibitors, *β*-blockers, vasodilators, lipid-lowering agents, aspirin, or heparin.

### 2.1. Surgical Technique

Patients underwent their operations on cardiopulmonary bypass with routine ascending-aorta and right-atrial cannulation. The core temperature was allowed to drift to 32°C, and aortic venting was performed. Myocardial protection was provided with antegrade and intermittent retrograde cold blood cardioplegia. Distal and proximal anastomoses were completed in a single aortic cross-clamp period.

### 2.2. Blood Sampling and Analysis

Blood samples were collected at the beginning of the study, before the administration of Melatonin or placebo (*T1*), after one-month administration of Melatonin or placebo just before operation (*T2*), when the aortic cross-clamp was placed (*T3*), and 45 minutes after the operation (*T4*).

Plasma concentrations of Melatonin were measured in duplicate by using Enzyme-Linked Immunoassay (IBL International, Germany). The intra-assay coefficient of variation was 6.7%. 

Melatonin levels in serum samples of (*T1*) and (*T2*) were measured with an enzyme immunoassay for the quantitative determination in human serum kit (IBL International, Hamburg, Germany).

Blood samples were collected from the central venous line before the aortic cross-clamp was placed (*T3*) and at 45 minutes after the operation (*T4*). 

Ten mL of blood was collected from each subject in a sterile glass tube containing 100 IU of preservative-free heparin (Sigma, St. Louis, MO, USA) at each of the time points described. 

Two mL of the each blood sample was immediately centrifuged at 4000 rpm for 10 minutes and then were aliquot and stored at −70°C in average for 3 months in order to analyze all samples simultaneously in the same procedure conditions. 8 mL of blood sample was used to isolate peripheral blood mononuclear cells (PBMCs) using the standard Ficoll-Hypaque (Histopaque, Sigma) density gradient centrifugation method. PBMCs were washed with PBS and immediately placed in –70°C and frozen for later analysis. Once PBMCs for all 30 subjects were collected, after prepare nuclear extraction by use of active motif kit, Nrf2 protein analysis was performed using TransAM Nrf2 kits (Active Motif, Carlsbad, CA, USA) which is an ELISA-based assay with Nrf2alpha-specific antibodies.

Protein total of both samples of (*T3*) and (*T4*) was measured by ZiestChem total protein kit which is based on Biuret method.

### 2.3. Statistical Analysis

Statistical analysis was performed with SPSS statistical software (version 11.0; SPSS, Chicago, IL, USA). The results were analyzed to verify the normality of distribution using the test of Kolmogorov-Smirnov. Continuous variables were expressed as the mean ± SD, and differences were evaluated statistically with the Student's *t*-test for independent samples. *P* value < 0.05 was considered statistically significant.

## 3. Results and Discussion

The patients demographic data are summarized in Table 1. Melatonin group (Group I) consisted of 14 men and 1 women, with a mean age of 58.1 ± 9.8 years (range, 42–75 years). Placebo group (Group II) consisted of 12 men and 3 women, with a mean age of 60.1 ± 9.2 years (range, 41–75 years). The 2 groups were not significantly different with respect to sex, age, additional diseases (hypertension, diabetes mellitus), or ejection fraction. None of the patients had acute myocardial infarction.

Tables 2 and 3 summarize the perioperative and postoperative results of the patients.

At the beginning of the study, there were no significant different levels of Melatonin between 2 groups of patients while after 1-month Melatonin treatment the preoperative plasma levels of Melatonin (*T2*) were substantially higher in Melatonin group than in placebo group. 

NRF2/protein total ratio was also significantly higher in Melatonin group than in placebo group (*P* < 0.05) (Table 2). Aortic cross-clamp and cardiopulmonary bypass times for the 2 groups were similar (Table 3).

In the current study, we showed significant increase in Nrf2 levels in Melatonin-treated group compared with controls after CABG operation. The CABG surgery represents an oxidative stress status with possible harmful effects for the patients. The CHD patients are particularly prone to oxidative damage because of their reduced antioxidant defense [24, 25]. In the current study, we tested for the first time whether Melatonin would induce Nrf2 as a master transcription factor for management of the oxidative stress in patients undergoing CABG surgery.

Melatonin can control oxidative stress status through different mechanisms. This molecule is a direct free radical scavenger; it induces antioxidative enzymes and inhibits proxoxidative enzyme; Melatonin stabilizes cell membrane and increases the efficiency of mitochondrial oxidative phosphorylation [26]. Besides, several clinical and experimental studies have confirmed that Melatonin has low toxicity [27]. 

The mechanisms by which Melatonin induced the expression of Nrf2 are not investigated in this study. However, based on previous studies, at least four major mechanisms are involved in the actions of Melatonin, including binding to membrane receptors [28], binding to nuclear receptors [29], interactions with cytoplasmic proteins [30], and direct scavenging free radicals [31]. 

The mechanisms that underlie the effects of preoperative Melatonin on postoperative outcomes are unclear, but several experimental studies have demonstrated the beneficial effects of Melatonin in several conditions associated with IRI.

Salie et al. [32] reported that Melatonin protects rat ventricular myocytes injury against IRI, through inhibition of reactive oxygen species generation and intracellular Ca^2+^ accumulation.

Tan et al. [33] found that Melatonin infusion throughout the period of occlusion and after reopening of the coronary artery in a Langendorff rat heart model significantly reduced both premature ventricular contractions and the ventricular fibrillation.

Lagneux et al. [34] showed that Melatonin's free radical scavenging activity reduces infarct volume after IRI and restores cardiac function. Additional investigations [35–38] have confirmed the ameliorative effects of pharmacologic doses of Melatonin on cardiac tissue injury and function after IRI.

The beneficial effects of Melatonin may derive from its stimulatory effect on antioxidative enzymes including superoxide dismutase, glutathione peroxidase, glutathione reductase, and glucose-6 phosphate dehydrogenase and its inhibitory action on the prooxidative inducible NO synthase [39].

Dobsak et al. [40] showed that Melatonin can suppress apoptosis and scavenge the peroxyl radical in a rat experimental model of ischemia-reperfusion injury.

In the present research, we found that Nrf2-ARE pathway could be further upregulated by the administration of Melatonin in patients undergoing CABG.

In similar researches regarding the association of Melatonin and Nrf2-ARE pathway, Crespo et al. [41] examined the effects of Melatonin on oxidative stress and Nrf2-ARE pathway in an animal model of fulminant hepatic failure. They found that Melatonin treatment resulted in an increased protein expression of Nrf2 in the cytoplasm and the nucleus and played a potential hepatoprotective role in fulminant hepatic failure, partly mediated through the abrogation of oxidative stress and the prevention of the decreased activity of antioxidant enzymes via the Nrf2-ARE pathway. Similarly, as mentioned by Tripathi and Jena [42] in their literature, they investigated the influence of Melatonin on urotoxicity associated with cyclophosphamide chemotherapy in patients with cancer and found that Melatonin treatment favorably changed Nrf2 expression, which appeared to be, at least in part, responsible for observed protection against cyclophosphamide-induced urotoxicity. 

## 4. Conclusions

 In the present research, we found that administration of Melatonin in patients undergoing CABG can upregulate the activity of Nrf2-ARE pathway with many potential beneficial effects. Further studies with larger sample size and over a longer period of time in patients are warranted to delineate the role of Melatonin in reducing postoperative oxidative stress in response to surgery. The mechanisms of Melatonin's effect in activation of Nrf2 and regulation of some other transcription factors could be further investigated. 

## Figures and Tables

**Table 1:** Preoperative clinical data of the patients*.

Variable	Melatonin group (*n* = 15)	Placebo group (*n* = 15)	*P*
Male/female sex, *n*	14/1	12/3	NS
Age, *y*	58.1 ± 9.8	60.1 ± 9.2	NS
Hypertension, *n*	8	7	NS
Diabetes mellitus type I or II, *n*	4	4	NS
Acute myocardial infarction	0	0	NS
Ejection fraction, %	58.5 ± 7.2	56.5 ± 4.6	NS

*Data are presented as the mean ± SD, where indicated. NS: not statistically significant.

**Table 2:** The plasma levels of Melatonin and the intracellular levels of Nrf2 in different sampling session during the study.

Variable/sampling time	Group I (Melatonin)	Group II (placebo)	*P* ^†^	*P* ^‡^
Melatonin, pg/mL				
*T*1	2.11 ± 1.06	1.63 ± 0.88	NS	0.008 (Group I)
*T*2	15.2 ± 4.6	1.1 ± 0.59	<0.001	NS (Group II)
NRF2/protein total				
*T*3	0.2819 ± 0.15	0.2092 ± 0.07	<0.05	NS (Group I)
*T*4	0.2693 ± 0.09	0.1998 ± 0.04	<0.01	NS (Group II)

*Data are presented as the mean ± SD. Sampling times were before the administration of Melatonin or placebo (*T*1), operation, after one-month administration of Melatonin or placebo (*T*2) before the aortic cross-clamp was placed (*T*3), and 45 minutes (*T*4) after the operation.

^†^ *P* values for the difference between groups I and II at the same time point.

^‡^ *P* values determined by paired *t*-test for differences at consecutive time points between groups.

**Table 3:** Perioperative and postoperative data of the patients*.

Variable/sampling time	Group I (Melatonin)	Group II (placebo)	*P* ^†^	*P* ^‡^
Cross-clamp time, min	49.7 ± 18.4	42.7 ± 5.0	NS	NA
CPB time, min	65.0 ± 10.1	70.6 ± 8.7	NS	NA
Po2				
*T*3	349 ± 66.6	323 ± 44.4	NS	<0.001 (Group I)
*T*4	63.1 ± 14.1	42.4 ± 4.8	NS	<0.001 (Group II)
O2 sat				
*T*3	75.4 ± 11.2	79.7 ± 9.8	NS	NS
*T*4	71.4 ± 22.7	62.8 ± 15.2	NS	<0.05 (Group II)
Pco2				
*T*3	39.2 ± 6.1	42.0 ± 220	NS	<0.05 (Group I)
*T*4	45.8 ± 8	45.7 ± 4.3	NS	NS
*T*3	28.8 ± 5.6	27.3 ± 5.6	NS	NS
*T*4	27.2 ± 2	27.3 ± 2.1	NS	NS

*Data are presented as the mean ± SD. Sampling times were preoperatively before the aortic cross-clamp was placed (*T*3) and at 45 min (*T*4) postoperatively. NS: not statistically significant; CPB: cardiopulmonary bypass; and NA: not applicable.

^†^ *P* values for the difference between groups I and II at the same time point.

^‡^ *P* values determined by paired *t*-test for differences at consecutive time points between groups.
